# Trends in antibacterial resistance among *Streptococcus pneumoniae *isolated in the USA: update from PROTEKT US Years 1–4

**DOI:** 10.1186/1476-0711-7-1

**Published:** 2008-01-11

**Authors:** Stephen G Jenkins, Steven D Brown, David J Farrell

**Affiliations:** 1 Mount Sinai Medical Center, Mount Sinai School of Medicine, New York, NY, USA; 2 Clinical Microbiology Institute, Wilsonville, OR, USA; 3 G.R. Micro Ltd, 7-9 William Road, London, UK

## Abstract

**Background:**

The increasing prevalence of resistance to established antibiotics among key bacterial respiratory tract pathogens, such as *Streptococcus pneumoniae*, is a major healthcare problem in the USA. The PROTEKT US study is a longitudinal surveillance study designed to monitor the susceptibility of key respiratory tract pathogens in the USA to a range of commonly used antimicrobials. Here, we assess the geographic and temporal trends in antibacterial resistance of *S. pneumoniae *isolates from patients with community-acquired respiratory tract infections collected between Year 1 (2000–2001) and Year 4 (2003–2004) of PROTEKT US.

**Methods:**

Antibacterial minimum inhibitory concentrations were determined centrally using the Clinical and Laboratory Standards Institute (CLSI) broth microdilution method; susceptibility was defined according to CLSI interpretive criteria. Macrolide resistance genotypes were determined by polymerase chain reaction.

**Results:**

A total of 39,495 *S. pneumoniae *isolates were collected during 2000–2004. The percentage of isolates resistant to erythromycin, penicillin, levofloxacin, and telithromycin were 29.3%, 21.2%, 0.9%, and 0.02%, respectively, over the 4 years, with marked regional variability. The proportion of isolates exhibiting multidrug resistance (includes isolates known as penicillin-resistant *S. pneumoniae *and isolates resistant to ≥ 2 of the following antibiotics: penicillin; second-generation cephalosporins, e.g. cefuroxime; macrolides; tetracyclines; and trimethoprim-sulfamethoxazole) remained stable at ~30% over the study period. Overall *mef*(A) was the most common macrolide resistance mechanism. The proportion of *mef*(A) isolates decreased from 68.8% to 62.3% between Year 1 and Year 4, while the percentage of isolates carrying both *erm*(B) and *mef*(A) increased from 9.7% to 18.4%. Over 99% of the *erm*(B)+*mef*(A)-positive isolates collected over Years 1–4 exhibited multidrug resistance. Higher than previously reported levels of macrolide resistance were found for *mef*(A)-positive isolates.

**Conclusion:**

Over the first 4 years of PROTEKT US, penicillin and erythromycin resistance among pneumococcal isolates has remained high. Although macrolide resistance rates have stabilized, the prevalence of clonal isolates, with a combined *erm*(B) and *mef*(A) genotype together with high-level macrolide and multidrug resistance, is increasing, and their spread may have serious health implications. Telithromycin and levofloxacin both showed potent *in vitro *activity against *S. pneumoniae *isolates irrespective of macrolide resistance genotype.

## Background

*Streptococcus pneumoniae *is a common causative pathogen in community-acquired respiratory tract infections (RTIs), including acute otitis media [1], acute bacterial exacerbations of chronic bronchitis [2], acute bacterial sinusitis [3], and community-acquired pneumonia [4]. It is also a major cause of bacteremia [5].

During the last decade, antimicrobial resistance has increased both in the USA [6-13] and worldwide [14-17]. In particular, resistance to penicillin and the macrolides has spread rapidly among isolates of *S. pneumoniae *[18]. Surveillance data have shown that, among *S. pneumoniae *isolates obtained from pediatric patients, the proportion exhibiting nonsusceptibility to penicillin increased each year after 1994, reaching 45% in 2000 [19]. There has also been a marked shift to high-level resistance to penicillin and cephalosporins among isolates of *S. pneumoniae*. Furthermore, several published studies and case reports (reviewed by Rzeszutek and colleagues [20]) have suggested a link between pneumococcal macrolide resistance and treatment failure (resulting in hospitalization) in patients with community-acquired RTIs.

Macrolide resistance among *S. pneumoniae *is mediated by two major mechanisms: methylation of ribosomal macrolide target sites, encoded by the *erm*(B) gene, and drug efflux, encoded by the *mef*(A) gene [18]. While *erm*(B)-mediated resistance predominates across much of the world, the dominant genotype in the USA is *mef*(A) [21]. *S. pneumoniae *isolates with both *erm*(B) and *mef*(A) genes have also been documented in the USA [21,22], and are typically multidrug resistant and clonal in nature [11,23]. These findings have raised concerns over the continued clinical utility of antibacterial agents, such as the β-lactams and macrolides, for the empiric treatment of many community-acquired RTIs.

PROTEKT US (**P**rospective **R**esistant **O**rganism **T**racking and **E**pidemiology for the **K**etolide **T**elithromycin in the **US**) – a longitudinal surveillance study – was initiated in 2000 to monitor resistance trends in *S. pneumoniae *and other common RTI pathogens in the USA [16]. A major aim of the program is to evaluate the activity of telithromycin, the first in a new class of antibacterial agents, and to compare its activity to that of other commonly used antibacterials. Data from the PROTEKT US study for 2000–2001 and 2001–2002 (Years 1 and 2) indicated a national prevalence of pneumococcal macrolide resistance of 31%, with rates approaching 40% in some southern regions of the country [10,24]. Data from 2000–2003 (Years 1–3) of the PROTEKT US study [13] demonstrated that the proportion of *S. pneumoniae *isolates exhibiting multidrug resistance has stabilized (31%). However, geographic variations remain and there is an increasing prevalence of isolates with both *erm*(B) and *mef*(A) genes, which is associated with high-level macrolide and multidrug resistance. The potential spread of macrolide and multidrug resistance is of serious concern and requires further monitoring. Telithromycin, however, continues to display potent *in vitro *activity against *S. pneumoniae*, including against isolates with the combined *erm*(B)+*mef*(A) genotype.

This paper reports on the phenotypic susceptibility and the distribution of macrolide resistance genotypes for *S. pneumoniae *isolates collected in Year 4 (2003–2004) of the PROTEKT US study; in addition, it provides an update on temporal and geographic trends in resistance patterns over the 4 years of the study.

## Methods

### Collection centers

The numbers of centers across the USA that contributed samples were: 207 in Year 1 (2000–2001), 241 in Year 2 (2001–2002), 247 in Year 3 (2002–2003), and 183 in Year 4 (2003–2004). For the purposes of geographic analysis, centers were assigned to one of six regions: Northwest (Alaska, Idaho, Montana, Oregon, Washington, Wyoming), Northeast (Connecticut, Delaware, Indiana, Maryland, Massachusetts, Michigan, New Jersey, New York, Ohio, Pennsylvania, Vermont, Washington, DC), North-central (Illinois, Iowa, Kansas, Minnesota, Missouri, Nebraska, North Dakota, South Dakota, Wisconsin), Southwest (Arizona, California, Colorado, Nevada, New Mexico, Utah), Southeast (Florida, Georgia, Kentucky, North Carolina, Puerto Rico, South Carolina, Virginia, West Virginia), and South-central (Alabama, Arkansas, Louisiana, Oklahoma, Tennessee, Texas).

### Bacterial isolates

Pathogenic respiratory tract isolates of *S. pneumoniae *were obtained from pediatric and adult outpatients with community-acquired RTIs (bacterial sinusitis, acute otitis media, pharyngitis, community-acquired pneumonia, acute bacterial exacerbations of chronic bronchitis, and acute exacerbations of chronic obstructive pulmonary disease). *S. pneumoniae *isolates cultured from material collected from hospitalized patients within 48 hours of admission were also included. Sources of isolates included blood, sputum, bronchoalveolar lavage, middle-ear fluid (sampled by tympanocentesis), nasopharyngeal swabs or aspirates, and sinus aspirates. Patients with cystic fibrosis and those with nosocomial RTIs were excluded from the study, as were strains originating from existing banked collections and duplicate strains. The following demographic data were collected: age and sex of patient, infection type, culture source, in-/outpatient status, specimen accession number, and date of sample collection. Details of the methods for isolate storage, transportation, and identification have been reported previously [16].

### Antibacterial susceptibility testing

Antibacterial minimum inhibitory concentrations (MICs) were determined using the Clinical and Laboratory Standards Institute (CLSI) broth microdilution method [25] at a central laboratory (CMI, Wilsonville, OR, USA). CLSI MIC interpretive criteria were used to determine susceptibility [26]. Susceptibility of *S. pneumoniae *isolates to telithromycin was determined using breakpoints approved by both the CLSI and the US Food and Drug Administration (susceptible ≤ 1 μg/mL; intermediate 2 μg/mL; resistant ≥ 4 μg/mL) [26,27]. Multidrug-resistant *S. pneumoniae *were defined as isolates resistant to ≥ 2 of the following antibiotics: penicillin; second-generation cephalosporins, e.g. cefuroxime; macrolides; tetracyclines; and trimethoprim-sulfamethoxazole.

### Genotyping

Erythromycin-resistant (MIC ≥ 1 μg/mL) pneumococcal isolates were analyzed for the presence of *erm*(B), *erm*(A) subclass *erm*(TR), and *mef*(A) macrolide resistance genes. Isolates in Year 1 were analyzed using a multiplex rapid-cycle polymerase chain reaction (PCR) with microwell-format probe hybridization, as described previously [28]; a Taqman^®^-based PCR assay was used in Years 2–4 [29].

## Results

### Isolates

In total, 39,495 *S. pneumoniae *isolates were collected in the PROTEKT US study from 2000 to 2004 (Year 1: 10,103; Year 2: 10,012; Year 3: 10,886; and Year 4: 8,494). Patient demographics and the culture source of *S. pneumoniae *isolates were similar in each year of the study (Table 1). Overall for Years 1–4 combined, most isolates of *S. pneumoniae *were collected from patients aged 15–64 years (42%) and approximately 50% of patients were hospitalized. The most common source of isolates was sputum (40.2%), followed by blood (28.7%) and bronchoalveolar lavage (10.6%).

**Table 1:** Patient demographics and culture source of *Streptococcus pneumoniae *isolates collected during the PROTEKT US study Years 1–4 (2000–2004)

	No. of isolates (%)			
	
	Year 1 (n = 10,103)	Year 2 (n = 10,012)	Year 3 (n = 10,886)	Year 4 (n = 8,494)
Age (years)				
0–2	1,822 (18.0)	1,556 (15.5)	1,587 (14.6)	1,213 (14.3)
3–14	1,105 (11.0)	1,125 (11.2)	1,324 (12.2)	973 (11.5)
15–64	4,144 (41.0)	4,058 (40.5)	4,617 (42.4)	3,751 (44.2)
> 64	2,761 (27.3)	3,067 (30.6)	3,091 (28.4)	2,378 (28.0)
NR	271 (2.7)	206 (2.1)	267 (2.5)	179 (2.1)
Gender				
Male	5,735 (56.8)	5,678 (56.7)	6,013 (55.2)	4,755 (56.0)
Female	4,252 (42.1)	4,218 (42.1)	4,757 (43.7)	3,575 (42.1)
NR	116 (1.1)	116 (1.2)	116 (1.1)	164 (1.9)
Source*				
Blood	3,220 (31.9)	2,717 (27.1)	3,016 (27.7)	2,398 (28.2)
BAL	968 (9.6)	1,019 (10.2)	1,197 (11.0)	993 (11.7)
Sputum	3,736 (37.0)	4,115 (41.1)	4,595 (42.2)	3,428 (40.4)
Sinus	388 (3.8)	390 (3.9)	506 (4.6)	446 (5.3)
Ear	858 (8.5)	620 (6.2)	813 (7.5)	556 (6.5)
MEF	68 (0.7)	69 (0.7)	91 (0.8)	88 (1.0)
Nasopharyngeal	632 (6.3)	573 (5.7)	649 (6.0)	540 (6.4)
Throat	48 (0.5)	28 (0.3)	--	6 (0.1)
Patient status				
Inpatient	4,612 (45.6)	5,359 (53.5)	6,068 (55.7)	4,599 (54.1)
Outpatient	5,287 (52.3)	4,349 (43.4)	4,818 (44.3)	3,776 (44.5)
NR	204 (2.0)	304 (3.0)	--	119 (1.4)

*Where isolate source was specified

BAL, bronchoalveolar fluid; MEF, middle-ear fluid; NR, not recorded.

### Antibacterial resistance patterns

High-level penicillin resistance (MIC ≥ 2 μg/mL) decreased during Years 1–4 (Year 1, 26.3%; Year 4, 16.5%) (Table 2) and was associated with a concomitant rise in intermediate-level resistance to this antimicrobial (MIC 0.12–1 μg/mL) over the study period (Year 1, 12.5%; Year 4, 20.0%). The incidence of erythromycin resistance was similar across all study years (29.3% overall). In general, resistance rates for other antimicrobials were also stable, with the exception of trimethoprim-sulfamethoxazole, which decreased from 33.9% in Year 1 to 24.1% in Year 4. The prevalence of resistance to telithromycin and levofloxacin was low; in each year, > 99% and > 98% of *S. pneumoniae *isolates were susceptible to telithromycin and levofloxacin, respectively.

**Table 2:** Rates of resistance to various antibacterials among *Streptococcus pneumoniae *isolates collected during Years 1–4 (2000–2004) of the PROTEKT US study

Antibacterial	Year 1 (n = 10,103)	Year 2 (n = 10,012)	Year 3 (n = 10,886)	Year 4 (n = 8,494)
Penicillin				
MIC_50_/MIC_90 _(μg/mL)	0.06/4	0.06/2	≤ 0.03/2	≤ 0.03/2
MIC range (μg/mL)	0.06–16	0.06–16	≤ 0.03–16	≤ 0.03–16
Resistant (% of isolates)	26.3	21.2	20.2	16.5
Intermediate (% of isolates)	12.5	14.2	15.3	20.0
Amoxicillin-clavulanate				
MIC_50_/MIC_90 _(μg/mL)	≤ 0.12/2	≤ 0.12/2	≤ 0.12/2	≤ 0.12/2
MIC range (μg/mL)	≤ 0.12-≥ 8	≤ 0.12-≥ 8	≤ 0.12-≥ 8	≤ 0.12-≥ 8
Resistant (% of isolates)	4.4	3.5	4.0	4.1
Cefuroxime axetil				
MIC_50_/MIC_90 _(μg/mL)	≤ 0.12/8	≤ 0.12/8	0.25/8	0.25/4
MIC range (μg/mL)	≤ 0.12-≥ 16	≤ 0.12-≥ 16	0.25-≥ 16	≤ 0.12-≥ 16
Resistant (% of isolates)	28.8	24.1	23.0	20.4
Erythromycin				
MIC_50_/MIC_90 _(μg/mL)	0.12/16	≤ 0.06/16	0.12/64	≤ 0.06/256
MIC range (μg/mL)	≤ 0.06-≥ 256	≤ 0.06-≥ 256	≤ 0.06-≥ 256	≤ 0.06-≥ 256
Resistant (% of isolates)	31.0	27.9	29.2	29.1
Clarithromycin				
MIC_50_/MIC_90 _(μg/mL)	0.06/16	≤ 0.03/16	≤ 0.03/32	≤ 0.03/128
MIC range (μg/mL)	≤ 0.03-≥ 256	≤ 0.03-≥ 256	≤ 0.03-≥ 256	≤ 0.03-≥ 256
Resistant (% of isolates)	30.7	27.5	28.7	28.9
Azithromycin				
MIC_50_/MIC_90 _(μg/mL)	0.12/32	0.12/32	0.12/≥ 256	0.12/256
MIC range (μg/mL)	≤ 0.03-≥ 256	≤ 0.03-≥ 256	≤ 0.03-≥ 256	≤ 0.03-≥ 256
Resistant (% of isolates)	31.0	27.7	28.9	28.9
Telithromycin				
MIC_50_/MIC_90 _(μg/mL)	≤ 0.015/0.5	≤ 0.015/0.25	≤ 0.015/0.5	≤ 0.015/0.5
MIC range (μg/mL)	≤ 0.015–8	≤ 0.015–4	≤ 0.015–4	≤ 0.015-≥ 4
Resistant (% of isolates)	0.04	0.02	0.01	0.01
Levofloxacin				
MIC_50_/MIC_90 _(μg/mL)	1/1	1/1	1/1	1/1
MIC range (μg/mL)	≤ 0.12–128	≤ 0.12-≥ 256	≤ 0.12–128	≤ 0.12-≥ 256
Resistant (% of isolates)	0.8	1.1	0.8	1.0
Tetracycline				
MIC_50_/MIC_90 _(μg/mL)	0.25/≥ 8	0.25/≥ 8	0.25/≥ 8	0.25/≥ 8
MIC range (μg/mL)	≤ 0.06-≥ 8	≤ 0.06-≥ 8	≤ 0.06-≥ 8	≤ 0.06-≥ 8
Resistant (% of isolates)	15.9	14.9	14.8	14.6
Trimethoprim-sulfamethoxazole				
MIC_50_/MIC_90 _(μg/mL)	≤ 0.25/≥ 8	≤ 0.25/≥ 8	≤ 0.25/≥ 8	≤ 0.25/≥ 8
MIC range (μg/mL)	≤ 0.25-≥ 8	≤ 0.25-≥ 8	≤ 0.25-≥ 8	≤ 0.25≥ 8
Resistant (% of isolates)	33.9	28.2	26.8	24.1

MIC, minimum inhibitory concentration.

**Figure 1:**
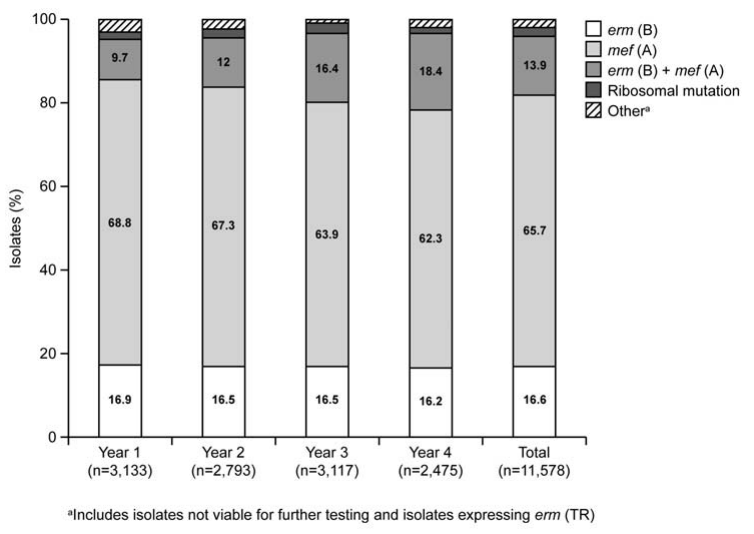
Geographic distribution of rates of penicillin and erythromycin nonsusceptibility in *Streptococcus pneumoniae *isolates (n = 39 495) collected during the 4 years of the PROTEKT US study (2000–2004). North-central: Illinois, Iowa, Kansas, Minnesota, Missouri, Nebraska, North Dakota, South Dakota, Wisconsin; Northeast: Connecticut, Delaware, District of Columbia, Indiana, Maryland, Massachusetts, Michigan, New Jersey, New York, Ohio, Pennsylvania, Vermont; Northwest: Alaska, Idaho, Montana, Oregon, Washington, Wyoming; South-central: Alabama, Arkansas, Louisiana, Oklahoma, Tennessee, Texas; Southeast: Florida, Georgia, Kentucky, North Carolina, Puerto Rico, South Carolina, Virginia, West Virginia; Southwest: Arizona, California, Colorado, Nevada, New Mexico, Utah. Ery^I^, erythromycin intermediate; Ery^R^, erythromycin resistant; Pen^I^, penicillin intermediate; Pen^R^, penicillin resistant.

Combined data for Years 1–4 confirm that geographic variations in the prevalence of pneumococcal resistance to either penicillin or erythromycin exist across the USA (Figure 1). For Year 4, resistance to penicillin was highest in the South-central (n = 1,333), Southeast (n = 1,025), and North-central (n = 1,999) regions (19.1% of isolates for each region) and lowest in the Southwest (n = 927; 10.0%). Penicillin resistance decreased in all regions between Years 1 and 4. The greatest reductions in penicillin resistance were observed in the Southeast (Year 1, 36.4%; Year 4, 19.1%), followed by the Southwest (Year 1, 27.0%; Year 4, 10.0%) and South-central (Year 1, 32.5%; Year 4, 19.1%) regions. In contrast, intermediate-level penicillin resistance increased in most regions (except in the Southwest region). In all regions, resistance rates for erythromycin were higher than those for penicillin. In Year 4, resistance to erythromycin was highest in the South-central region (39.4%) and lowest in the Southwest region (17.0%). Erythromycin resistance rates decreased from Year 1 to Year 4 in the Southeast (Year 1, 40.2%; Year 4, 32.6%) and Southwest (Year 1, 29.3%; Year 4, 17.0%) regions, and remained stable or increased slightly in all other regions.

The prevalence of *S. pneumoniae *isolates that demonstrated resistance to both penicillin and erythromycin decreased over the study period (Year 1, 20.4%; Year 4, 13.3%) (Table 3). Decreases in the proportion of isolates resistant to both drugs were observed between Years 3 and 4 in all regions, but were most apparent in the South-central (Year 3, 21.9%; Year 4, 15.3%) and Southeast (Year 3, 19.6%; Year 4, 15.3%) regions.

**Table 3:** Prevalence of co-resistance to penicillin and erythromycin among *Streptococcus pneumoniae *isolates collected during the PROTEKT US study Years 1–4 according to US region

	Proportion of isolates (n [%])			
	
Region	Year 1	Year 2	Year 3	Year 4
North-central	446 (21.2)	385 (17.7)	431 (17.8)	321 (16.1)
Northeast	606 (16.3)	428 (13.5)	471 (13.9)	336 (12.1)
Northwest	55 (13.0)	73 (13.7)	66 (12.1)	42 (9.7)
South-central	381 (26.2)	309 (23.2)	378 (21.9)	204 (15.3)
Southeast	310 (29.2)	309 (22.2)	282 (19.6)	157 (15.3)
Southwest	264 (19.6)	205 (14.5)	152 (11.2)	70 (7.6)
USA total	2,061 (20.4)	1,709 (17.1)	1,780 (16.4)	1,130 (13.3)

The prevalence of multidrug resistance among *S. pneumoniae *remained relatively stable over the study period, particularly for Years 2–4, at approximately 30% of isolates (Table 4). Some decreases in the proportions of multidrug-resistant isolates were observed in southern regions (Year 1/Year 4: Southeast, 44.6%/31.4%; Southwest, 35.7%/17.4%). In Year 3, the prevalence of multidrug resistance in the Southwest was 22.4%; this decreased to 17.4% by Year 4.

**Table 4:** Prevalence of multidrug resistance among *Streptococcus pneumoniae *isolates collected during the PROTEKT US study Years 1–4 (2000–2004) according to US region

	Proportion of isolates (n [%])			
	
Region	Year 1	Year 2	Year 3	Year 4
North-central	727 (34.4)	651 (29.8)	730 (30.1)	599 (30.0)
Northeast	1,064 (28.7)	750 (23.5)	883 (26.0)	670 (24.1)
Northwest	108 (25.6)	120 (22.6)	124 (22.8)	95 (21.9)
South-central	635 (43.6)	498 (37.4)	660 (38.2)	506 (38.0)
Southeast	474 (44.6)	491 (35.3)	487 (33.9)	322 (31.4)
Southwest	481 (35.7)	394 (27.9)	306 (22.4)	161 (17.4)
USA total	3,489 (34.5)	2,904 (28.9)	3,190 (29.3)	2,353 (27.7)

**Figure 2:**
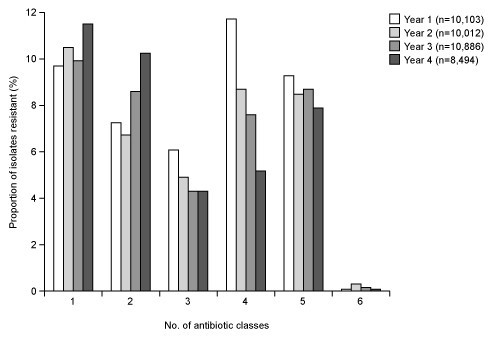
Prevalence of resistance to 1, 2, 3, 4, 5, or 6 antibacterial agents^a ^among *Streptococcus pneumoniae *isolates collected during the PROTEKT US study Years 1–4 (2000–2004). ^a^Penicillin (MIC ≥ 2 μg/mL), erythromycin (MIC ≥ 1 μg/mL), cefuroxime (MIC ≥ 4 μg/mL), tetracycline (MIC ≥ 8 μg/mL), trimethoprim-sulfamethoxazole; MIC ≥ 4 μg/mL), and levofloxacin (MIC ≥ 8 μg/mL).

The temporal trends in the prevalence of resistance to between 1 and 6 antibacterial agents are shown in Figure 2. During Years 1–4, there was no increasing trend towards resistance of isolates to a greater number of antibacterial agents. The prevalence of isolates resistant to 3, 4, or 5 classes of antibacterial, however, was high and approximately 8% of isolates demonstrated resistance to 5 classes of antibiotic.

### Resistance mechanisms

**Figure 3:**
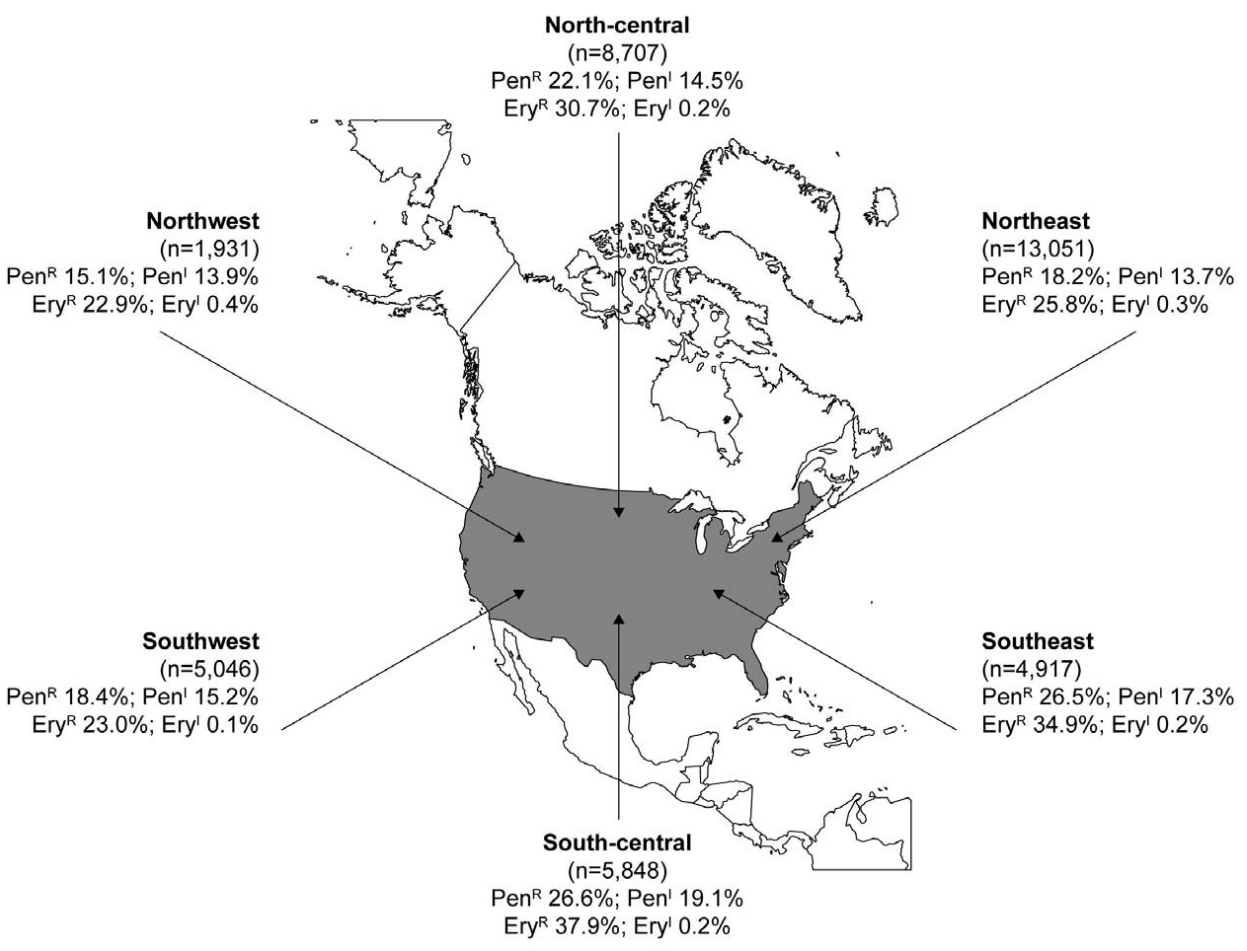
Distribution of resistance genes among macrolide-resistant *Streptococcus pneumoniae *isolates (n = 11 578) collected during the PROTEKT US study Years 1–4 (2000–2004).

Data from Years 1–4 show that *mef*(A)-encoded resistance was consistently the most commonly expressed genotype (65.7%) (Figure 3). However, across the 4-year study period, the proportion of macrolide-resistant isolates carrying both the *mef*(A) and *erm*(B) genes increased dramatically. In Year 1, 9.7% of macrolide-resistant isolates possessed both the *mef*(A) and *erm*(B) genes; this proportion increased to 12.0% in Year 2, rising to 16.4% in Year 3 and 18.4% in Year 4. Over the same period, isolates exhibiting *mef*(A) alone decreased and those exhibiting *erm*(B) remained stable. Overall, for Years 1–4 > 99 % (1,605/1,615 of the *erm*(B)+*mef*(A) isolates) were multidrug resistant (455/456 [99.8%] in Year 4). Analysis of the *in vitro *activity of a range of antibacterials against *mef*(A)-positive isolates found that all demonstrated higher than previously reported levels of resistance to erythromycin (MIC_90 _16 μg/mL; MIC mode 16 μg/mL). MIC_90 _values against these strains were also high for azithromycin (≥ 16 μg/mL; MIC mode 8 μg/mL) and clarithromycin (≥ 8 μg/mL; MIC mode μg/mL) (Table 5). By contrast, telithromycin retained strong activity against *mef*(A)-positive strains, with MIC_90 _values of 0.5 μg/mL (MIC mode 0.12 μg/mL).

**Table 5:** *In vitro *activity of selected antibacterials against erythromycin-resistant *Streptococcus pneumoniae *isolates positive for *mef*(A)

	Year 1 (n = 2,157)		Year 2 (n = 1,881)		Year 3 (n = 2,029)		Year 4 (n = 1,543)	
	
Antibacterial	MIC_90 _(μg/mL)	Susceptibility (%)	MIC_90 _(μg/mL)	Susceptibility (%)	MIC_90 _(μg/mL)	Susceptibility (%)	MIC_90 _(μg/mL)	Susceptibility (%)
Penicillin	4	12.3	4	13.0	4	14.3	4	19.0
Amoxicillin-clavulanate	≥ 8	71.8	4	78.8	4	82.9	2	90.7
Cefuroxime	8	23.4	8	28.8	8	34.4	8	44.5
Trimethroprim-sulfamethaxazole	≥ 8	12.4	≥ 8	17.1	≥ 8	19.0	≥ 8	27.6
Tetracycline	≥ 8	71.7	≥ 8	71.4	≥ 8	76.5	≥ 8	81.2
Erythromycin	16	0	16	0	16	0	16	0
Azithromycin	32	0	32	0.2	16	0	32	0
Clarithromycin	16	0.1	8	0.4	8	0.1	16	0.1
Levofloxacin	1	99.0	1	97.7	1	98.6	1	98.6
Telithromycin	1	99.1	0.5	99.9	0.5	99.9	0.5	99.9

MIC, minimal inhibitory concentration.

## Discussion and Conclusion

As part of the PROTEKT US study we have analyzed a large dataset for *S. pneumoniae *isolates sampled from across the USA. This has resulted in the generation of valuable information regarding temporal and geographic changes in antibacterial resistance patterns over the 4-year period from 2000 to 2004.

The latest data collected between 2003 and 2004 (Year 4) confirm the results of previous reports indicating that the prevalence of pneumococcal penicillin resistance across the USA appears to be stable or is decreasing, whilst intermediate penicillin resistance is increasing slightly [13,30]. There are a number of possible reasons why pneumococcal resistance to penicillin and some other antibacterials (e.g., trimethoprim-sulfamethoxazole) may have stabilized or begun to decrease in the USA [30]. First, recent local [31] and national [32] campaigns to promote appropriate antibacterial prescribing may have exerted a downward pressure on resistance rates as this factor is one of the most important drivers of resistance in community-acquired infections [33]. Another factor may be the introduction in 2000 of the pneumococcal conjugate vaccine (PCV7) for routine immunization of infants; resistance rates have traditionally been highest among the pediatric population and the use of PCV7 has been shown to decrease pneumococcal resistance not only among children but also in the population as a whole, via a herding effect [34]. A third factor may be the introduction of fluoroquinolones as a treatment for respiratory tract infections in adults. As the use of these agents has increased, it is possible that use of other more traditional agents may have declined, thus reducing associated rates of resistance.

Although the overall levels of *in vitro *penicillin-nonsusceptibility in *S. pneumoniae *may be a cause for concern, the clinical importance of this phenomenon in the management of pneumococcal pneumonia has been questioned [35-37]. There is no evidence of widespread clinical failures among respiratory infections caused by *S. pneumoniae *strains classified as penicillin-resistant *in vitro*. Moreover, in respiratory infections documented as being due to resistant pneumococci, the infecting *S. pneumoniae *strain generally exhibits low-level *in vitro *resistance (penicillin MIC 1–2 μg/mL); in these cases, the infection can usually be successfully treated using high doses of β-lactam antibiotics [38]. Nevertheless, careful monitoring of penicillin resistance rates should continue, especially since reports of *S. pneumoniae *strains with high-level penicillin resistance (MIC ≥ 8 μg/mL) have appeared recently [39,40]. Furthermore, there is clear evidence that infections of the central nervous system caused by penicillin-resistant *S. pneumoniae *strains can be associated with the failure of β-lactam therapy [38,41]. Consideration of the prevalent rates of penicillin resistance among *S. pneumoniae *is therefore important in the management of such infections.

In common with recent reports from the USA and other countries, macrolide resistance over the 4 years of PROTEKT US exceeded penicillin resistance in all US regions [13,16,17,30,42]. The macrolide resistance rate reported here for Year 4 was similar to those found for Years 1–3 (approximately 30%), suggesting that levels may have plateaued [12,13].

In this study, the proportion of isolates in Year 4 exhibiting resistance to both penicillin and erythromycin decreased compared with previous years; this downward trend was also observed over Years 1–3 of the PROTEKT US study [13]. As noted in other recent surveillance studies [7,10-13,43], data for Years 1–4 of PROTEKT US showed considerable regional variation in the rates of resistance to penicillin and erythromycin across the USA.

Macrolide resistance mediated by *erm*(B) has typically been associated with high-level resistance (MIC_90 _values of ≥ 64 μg/mL), while *mef*(A)-mediated resistance has historically been characterized by lower-level resistance (MIC_90 _values of 4–8 μg/mL) [15,44]. The predominant mechanism of pneumococcal macrolide resistance in the USA is mediated by *mef*(A) [21]. However, the latest PROTEKT US data presented here confirm that the prevalence of the *mef *(A) genotype is decreasing and that clones expressing both *erm*(B) and *mef*(A) genes are increasing in prevalence. Of additional importance, the *mef*(A)-positive isolates were found to exhibit levels of macrolide resistance that were higher (MIC mode 16 μg/mL) than those reported in previous surveillance studies (4–8 μg/mL) [15,43]. This may impact on the ability of the macrolides to eradicate such strains from the sites of infection in patients with community-acquired RTIs.

Molecular epidemiology studies undertaken as part of PROTEKT US have shown that, of the *erm*(B)+*mef*(A) isolates analyzed, > 90 % are clonally related to the multidrug-resistant international Taiwan^19F-14 ^clonal complex 271 [12]. Since these strains show high-level macrolide and multidrug resistance, their spread across the USA represents a serious public health threat.

The introduction of the 7-valent pneumococcal vaccine (PCV7) in 2000 was intended to reduce the incidence of pneumococcal disease in children. Recent evidence suggests that this reduction has indeed occurred [19,45], with decreases of 58% in 2001 and 66% in 2002 in the number of invasive pneumococcal infections in children. However, the vaccine does not provide coverage against all *S. pneumoniae *serotypes. Most dual *erm*(B)+*mef*(A) isolates have been characterized as either serotype 19A or 19F and, although serotype 19F is represented in the PCV7 vaccine, 19A is not. As a result, incidence of the nonvaccine serotype 19A multidrug-resistant clone is proportionally higher in the pediatric population than in the past. According to recent surveillance data covering pneumococcal isolates collected in the USA, the prevalence of vaccine serotype 19F has decreased since introduction of PCV7, while that of nonvaccine serotype 19A has concomitantly increased [34,46]. Among *erm*(B)+*mef*(A) isolates collected for the PROTEKT US study in 2000–2001, serotype 19F was predominant over 19A (87% vs 8%); however, by 2003–2004, these two serotypes were of roughly equal prevalence (52% [19F] vs 46% [19A]) among *erm*(B)+*mef*(A) isolates [46].

Since the introduction of newer macrolide antibiotics, there has been a steady increase in macrolide resistance from year to year among pneumococci, which has been correlated with their consumption [47]. Macrolide antibacterials are commonly used for the empiric treatment of community-acquired RTIs; therefore, pneumococcal macrolide resistance is of increasing concern in the clinical setting [18]. In recent years, a number of reports have been published linking occurrences of macrolide treatment failure (often resulting in hospitalization with breakthrough bacteremia) to infection by macrolide-resistant strains of *S. pneumoniae *in patients with community-acquired RTIs. Clinical failures in patients treated with azithromycin and clarithromycin have been documented [48-51], and the number of reports appears to be increasing. It is notable that clinical failures have been reported in patients infected with pneumococcal strains expressing *mef*(A)-encoded macrolide resistance as well as in those with *erm*(B)-mediated resistance [52,53]. Expression of both *erm*(B) and *mef*(A) among *S. pneumoniae *isolates is strongly associated with the emergence of multidrug resistance. Almost all of the isolates (99.8%) expressing this dual mechanism of macrolide resistance in Year 4 of the study exhibited such resistance. Multidrug resistance has also been linked to an increased risk of clinical failure [54].

Telithromycin represents the first in a new class of antimicrobials – the ketolides. Telithromycin demonstrated potent *in vitro *activity against *S. pneumoniae *isolates, including *erm*(B)+*mef*(A) macrolide-resistant strains. The *in vitro *susceptibility of *S. pneumoniae *to telithromycin was very high in each of the study years, irrespective of the macrolide resistance mechanism. Overall, > 99% of macrolide-resistant *S. pneumoniae *isolates were susceptible to this agent. These data are in agreement with corresponding longitudinal data from the international PROTEKT Global study (1999–2003), which indicate that no significant change in telithromycin susceptibility has been observed since the launch of the drug in some European countries in 2000–2001 [55,56]. Currently, telithromycin is licensed in the USA for treating community-acquired pneumonia in adults; however, the most recent Infectious Diseases Society of America/American Thoracic Society consensus guidelines on the management of community-acquired pneumonia in adults [57] do not carry any recommendations regarding the clinical use of telithromycin. Recommendations will be finalized when further evaluation of the safety of telithromycin by the US Food and Drug Administration has been completed.

The findings in this study highlight the need for the judicious use of antimicrobials and the continued monitoring of pneumococcal resistance patterns – in particular, the spread of multiresistant clones. Physicians should take local or regional resistance patterns into consideration when choosing empiric antibacterial treatment for community-acquired RTIs.

In summary, antimicrobial resistance in *S. pneumoniae *appears to have stabilized in the USA. However, geographic variations remain, and the prevalence of isolates with the combined *erm*(B) and *mef*(A) genotype, associated with high-level macrolide resistance (MIC_50 _> 256 μg/mL) and multidrug resistance, continues to increase. Telithromycin retains potent *in vitro *activity against *S. pneumoniae*, including isolates with the combined *erm*(B)+*mef*(A) macrolide resistance genotype.

## Competing interests

It may appear that SGJ has a competing interest as it relates to this manuscript as he was an employee of Aventis Pharmaceutical, the sponsor of the PROTEKT project, from May of 2001 until August of 2002 and has served as a consultant as well as a member of the company's Speakers Bureau at various times since leaving the company.

SB has received contract funding, expense reimbursements, and honoraria from sanofi-aventis.

DJF has received research grants and consultancy fees from sanofi-aventis related to telithromycin research, publications, and presentations. Sanofi-aventis are financing the production of this manuscript.

## Authors' contributions

SGJ contributed to the analysis and interpretation of data related to this manuscript and provided clinical isolates to the project for testing. In addition, SGJ was involved in revising the manuscript critically for important intellectual content.

SB actively participated in the overall design and coordination of the study, collection of data, review of the manuscript, and has granted final approval for the publication of the manuscript.

DJF and colleagues at GR Micro Limited undertook the laboratory testing, data collection and analysis, and drafted the paper.
